# Evaluation of the vulnerability to public health events in the Guangdong-Hong Kong-Macao Greater Bay Area

**DOI:** 10.3389/fpubh.2022.946015

**Published:** 2022-09-09

**Authors:** Wenjing Cui, Jing Chen, Huawen Shen, Yating Zhang, Shuting Liu, Yiting Zhou

**Affiliations:** ^1^College of Geosciences and Tourism Management, Hanshan Normal University, Chaozhou, China; ^2^China Center for Special Economic Zone Research Shenzhen University, Shenzhen, China; ^3^Faculty of International Tourism and Management, City University of Macau, Macau SAR, China; ^4^The NO.1 Middle School of Suixi County, Zhanjiang, China; ^5^School of management, Guangzhou College of Commerce, Guangzhou, China

**Keywords:** public health event, urban agglomeration, vulnerability, entropy method, Guangdong-Hong Kong-Macao Greater Bay Area (GBA)

## Abstract

With the continuous improvement in the integration of urban agglomeration, a multi-functional, socialized, and complex dynamic system, effective prevention and control of emergent public health events have become increasingly important. Based on the Public-Health Vulnerability-Assessment-System of Urban Agglomeration (PVUA), the temporal and spatial differentiation characteristics of vulnerability in the Guangdong-Hong Kong-Macao Greater Bay Area (GBA) for the period of 2015-2019 are explored, and the vulnerable cities to public health events are identified in this area. The results can be summarized as follows: (1) The overall vulnerability to public health events in GBA decreases in the investigated period. (2) In the temporal dimension, accompanied by social and economic development, the sensitivity to public health events increases in GBA, and the coping capacity change from stable fluctuation to rapid improvement. (3) From the spatial dimension, the sensitivity level in GBA is low in the west, relatively high in the middle, and high in the southeast; the coping capacity is high in the southeast and low in the northwest; the collaborative governance capacity presents a spatial pattern of being low in the south and high in the north. (4) In the period of study, the vulnerability to public health events in Guangzhou and Jiangmen is stable at the lowest level, while that in Zhaoqing, Foshan, and Hong Kong SAR (Special Administrative Region) gradually reduces; the vulnerability in Shenzhen, Zhuhai, and Dongguan is fluctuating, and that in Huizhou, Zhongshan, and Macao SAR is continually maintained at a higher and the highest level.

## Introduction

In the 21st century, with its accompanying economic development, the loss caused by major public health emergencies has been increasing yearly. Public health events refer to the epidemic of infectious diseases and mass diseases with unknown causes that have (likely) occurred, which has (likely) caused heavy losses to public health. In 2020, comprehensively promoting the construction of a healthy China was established as a critical task in China's 14th Five-Year Plan for national economic and social development and the vision goal of 2,035, for improving people's quality of life and raising the level of social construction. Therein, improving the mechanisms for monitoring, alerting, and effectively solving public health emergencies is significant for ensuring the overall promotion of a healthy China. In the same year, the General Secretary of the CPC Central Committee, Xi Jinping, stressed the importance of strengthening the prevention and control mechanisms of public health and improving the emergency management system of public health, preventing and coping with major public health emergencies.

Health policy needs to be informed by scientifically based evidence (1). To improve the capability to guarantee public security and achieve a new level of people's well-being, special studies on public health events have become a hot topic. Studies have focused on warning mechanisms and preventive measures (2, 3). It is worth mentioning that current public health events research concerns various types of public disasters, including complex epidemic events (4), the effect of online data on early warning of public health events (5), regional coping capacity and coping strategies (6–11), psychological crisis (12), communities, and production sites (13–20), evolution and guidance of public opinions (21–24), and the impact of public health events on regional economic development (25). All these strategies might be implemented to increase the capability to respond to public health threats and to adapt to dynamic threat changes. However, in most cases, these measures deal with threats in a specific city during a particular period. Notably, the dynamic public health security measurement framework applicable to the Chinese context has not yet been established and perfected. It states a severe gap in establishing the public health events model (**Gap 1**).

In disaster management, risk is often described as a function of hazard and vulnerability (26). Vulnerability results from a set of risks in threat exposure, threat materialization, and lack of defense to cope with the threat (27). The earliest vulnerability studies began with a single exogenous disturbance (28). In the early stage, the vulnerability was used to study the disaster-causing factors of natural disasters and describe the damage to objects or systems affected by external effects (28–30). Later, the method was extended to social and economic dimensions, forming the vulnerability system theory, which was mainly used to validate and explain social-environmental systems (31), poverty (32, 33), emergent events (34) et al. Application of social vulnerability indices in disaster management and identification of vulnerable people and regions are not novel (35). Globally, several studies have proposed variations of social vulnerability indices for use mainly in the context of disaster management, and only a small number for management of pandemics (36) or public health events. More than 2 years since the first SARS-CoV-2 infections were reported, the COVID-19 pandemic remains an acute global emergency in 2022. Since 2020, to meet WHO's goals: promote health, keep the world safe, and serve the vulnerable (37), meanwhile, to mitigate COVID-19 risk, several researchers developed vulnerability indexes for pandemics (35, 38–40), which expand the research content of the vulnerability.

In public health events, the cross-domain crisis has become the most characteristic and destructive new crisis form, which bears the risk of causing secondary and derivative hazards. The natural defects of structural obstacles limit the conventional urban crisis emergency system. It cannot effectively deal with the new situation and the new problems caused by the cross-domain crisis (41). City planning decisions affect city vulnerabilities (42). As the degree of dependence among different cities within urban agglomeration gradually increases, such cities are easily affected when an accident occurs in one city, resulting in cross-region casualties (43). Compared with single cities, the types and quantities of emergency supplies in urban agglomerations are relatively complete, and the rescue teams are rather professional. From this perspective, vulnerability to public health events in urban agglomerations refers to the disaster-causing tendency of the breakout and effects of public health events in the urban agglomeration system under multiple factors. It is used to measure the potential risks in node cities. Accordingly, based on the vulnerability assessment of public health events in urban agglomerations, exploring disaster prevention strategies and coordinated handling methodologies of crisis events is of great significance for reducing losses and protecting public health. Recently, trans-regional public security governance in urban agglomerations has become a significant focus of academia, and the concepts such as the conceptual model for regional comprehensive risk calculation (44), the planning optimization of traffic network of urban agglomeration (45), the integrated risk assessment model for multiple disasters (46), and an information-sharing system for public security interaction of urban agglomeration (47) have been proposed.

Currently, the studies on cross-regional public security coordination in urban agglomerations primarily focus on theoretical discussions, conceptual model construction, and policy analysis (34, 44, 48–50). Numerous studies have demonstrated that accurate measurement and management of vulnerability to public health events will effectively improve the ability of regions to respond to public health events. There is still a gap in universal vulnerability system research on public health events in urban agglomerations, hindering the implementation of risk analysis and the control of public health events in such areas (**Gap 2**).

At present, the spatial organization of urban agglomerations in China presents a new pattern of 5 + 9 + 6 consisting of five national, nine regional, and six regional urban agglomerations (51). Therein, Guangdong-Hong Kong-Macao Greater Bay Area (GBA) covers the cities with the best development potential in China. The developed foreign trade, free flow of capital, and talent elements inside and outside made GBA the critical node of the global value chain and supply chain. The comprehensive governance system of the GBA is confronted with complicated public health events threats due to its characteristics of crossing social systems, crossing legal systems, and multiple controls crossing administrative levels. The GBA is China's most open area and regional financial center, and its regional security development has essential economic, political, and social significance. Accordingly, scientific assessment of vulnerability to public health events in GBA plays a critical role in effectively improving the prevention, control, coping, and governance capacity of public health events in GBA and reducing the loss and impact caused by such public health events. Additionally, with all cites in urban agglomerations facing threat in public health hazard, it is essential that identifying vulnerability specificities of each cites is essential to minimize the damage caused by the outbreak (39). Understanding how and where vulnerable regions might be impacted can greatly help with effective allocation of resources during the different disaster management phases—prevention, preparedness, response, mitigation, recovery, and reconstruction (26). Though several studies have been conducted to investigate the regional differences in vulnerability, scarce studies have investigated the vulnerability topic within public health events in the GBA (**Gap 3**). Hence, GBA has been constructively selected as this study's research area.

Therefore, according to Gap 1–3, this research intends to fill the research gaps in GBA by employing the sensitivity-coping capacity-collaborative governance framework for establishing the PVUA dynamic evaluation system innovatively. GBA is adopted as the public health vulnerability empirical research object. The weights of 23 indices are determined by the entropy method, and the vulnerability level and temporal and spatial evolution characteristics of public health events in GBA are extensively explored to provide academic support for developing GBA into a safe bay area.

Preconcluding, the main research objective is to investigate public health vulnerability in GBA. The specific objectives may be outlined as follows:

1. *To elaborate a dynamic evaluation system for the PVUA by employing the sensitivity level-coping capacity level-collaborative governance level framework;*
2. *To explore the vulnerability level and temporal and spatial evolution characteristics of public health events in GBA;*
3. *Based on publicly available data, to identify the vulnerable cities in GBA by holistically respecting public health events conditions;*
4. *To formulate the risk-avoidance manners for public health events conditions using the weights of 23 indices related to public health events emergency interventions*.

This study's novelty is in the appraisal of public health vulnerability from the GBA from top-class international urban agglomeration in China. Theoretical and pragmatic contributions are also derived from this study's outcomes. The study builds the PVUA dynamic evaluation system and gives an in-depth comprehension of specific promotion strategies from actual vulnerability performance with a strong potential of advancing stakeholders' and city management department' capacity to actualize higher security level results in future action plans and strategies. Moreover, providing vulnerable ranking cites to policy makers to priorities resource allocation and provide strategies for creating safe GBA. The study proposes the PVUA evaluation system of implementing a proposed safe and sustainable urban agglomeration strategy for GBA, China, which could also be utilized as a tool or model by relevant stakeholders across the globe to objectively and systematically assess public health events emergency performance to allow for prompt preventive/amendatory mechanism.

The rest of the paper is organized as follows. In the next section, we discuss the representativeness of the study area and the reasonableness of the source data. Therein, we outline the data employed and the methods to caliber statistical data. And this is followed by arguments justifying the dynamic evaluation system for the PVUA, explaining how the sensitivity, coping capacity, and collaborative governance subsystems reflect different aspects of the potential facilitators and inhibitors of the public health events crisis across urban agglomerations. Then we depict the results of the various subsystems model estimated in GBA and consider which indices appear to contribute most to the subsystems crisis. The fifth section elucidated the vulnerability level data of cities in GBA in five different years, and the reasons for the crisis and the solutions are discussed. Our final section presents some insights and conclusions regarding the role played by the PVUA model in managing vulnerability to public health in urban agglomerations.

## Overview of study areas and data sources

**Figure 1:**
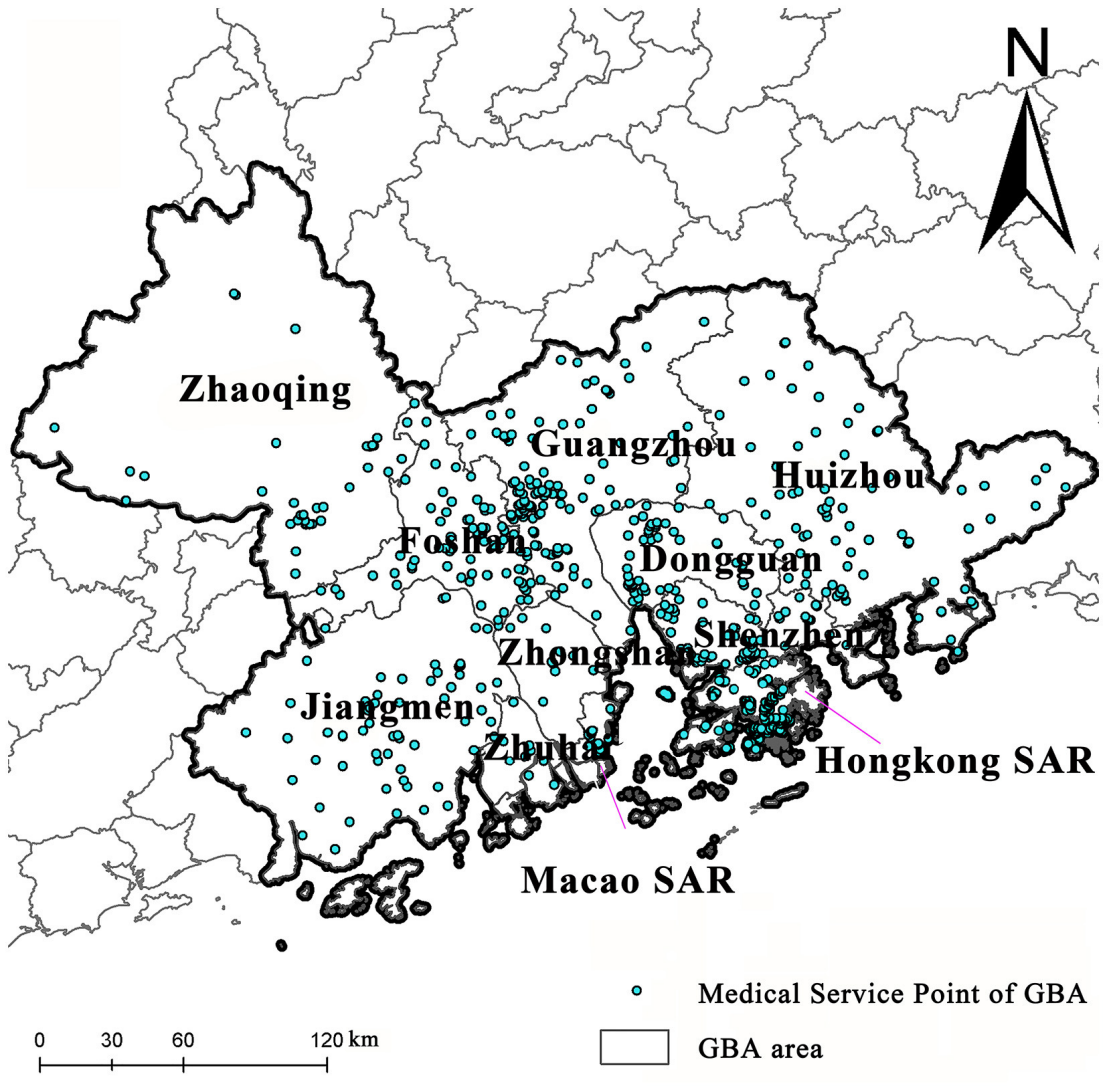
Research Are.

In the *Outline Development Plan for the Guangdong-Hong Kong-Macao Greater Bay Area* of 2019, it was explicitly pointed out to perfect the mechanism of effectively addressing emergencies, and construct an emergency coordination platform for GBA, to perfect the mechanism of effectively handling emergencies. The GBA is one of the regions with the highest degree of openness and the most substantial economic vitality in China (Figure 1). The agglomeration effect in GBA creates economic benefits and hidden dilemmas of crises. Its internal production factors are concentrated, its population and materials are spatial-mobile, and its administrative characteristics of 9+2 regional cooperation are unique. These, together with the differences between the different social and legal systems and the spatial heterogeneity of different customs zones, cause the research object of emergent public health events to exert a more significant impact with more widespread chain-reaction effects and crossing. During the prevention and control of the COVID-19 pandemic, GBA has cooperated closely in the connection and sharing of epidemic information, support and testing, material supply and guarantee, epidemic prevention technology exchange and patient treatment, and strict prevention of overseas import. As the research object, GBA is typical, representative, and demonstrative. Based on the five annual data sets in 2015-2019, the vulnerability to public health events in GBA is analyzed, and each city's vulnerability to public health events is rated to identify the difference in vulnerability to public health events effectively.

The geographic information data in this paper includes the data on prefecture-level city boundaries, road vector data, urban hospitals, and health service site data in the community. The geographic information data are derived from the database of the national geomatics system of China and Amap.com and are further extracted and calibrated to improve data accuracy. Meanwhile, socio-economic statistical data used in this study are mainly derived from the 2016–2020 China City Statistical Yearbooks, the 70 national demographic census data, annual government statistical bulletins, the Statistics and Census Service Bureau of Macao SAR, and the Census and Statistics Department of Hong Kong SAR. Some indices are obtained through statistical analysis of the original data. The statistical caliber of data had consistent unit conversions during the data collection process, such as government budget, social health expenditure, personal health expenditure, etc.

## Construction of the evaluation system for the PVUA

### The basic framework of the PVUA evaluation system

Quantitative measurement is an essential part of management science, and establishing conceptual ideas is a fundamental part of conducting quantitative measurements and a critical step in establishing the legitimacy of a concept (52). Identifying the characteristics of public health events in urban agglomerations is a prerequisite for establishing an effective disaster prevention and mitigation system. As the early research scale, Flanagan's social vulnerability index includes four domains—socioeconomic status, household composition and disability, minority status and language, and housing and transportation (53). In 2020, a study expanded the vulnerability concept of Flanagan and computed under different thematic domains, extending beyond social vulnerability to accommodate vulnerability related to the COVID-19 pandemic, and developed a socioeconomic vulnerability index (SVI). More specifically, five domains (socioeconomic, demographic, housing and hygiene, epidemiological, and health system) were adopted in SVI (54). WHO proposed the creation of a new dynamic, multi-hazards, evidence-based preparedness metric can gauge preparedness capacity dynamically and inform key action plans for improving capacities (37). On behalf of WHO Technical Working Group, a recent research established the dynamic preparedness metric (DPM) to measure a country's preparedness for health emergencies, which contains three main conceptual dimensions: hazard, vulnerability, and capacity (37).

In rapid economic growth, the security of public health incidents in urban agglomerations is characterized by obvious systematicity, diversity, derivation of crisis space, and the intersection of administrative management. These characteristics make the regional security system extremely vulnerable (29). Additionally, with the rapid economic and social development, urban agglomerations, as areas rich in medical resources and transportation facilities, have the advantages of high-quality medical resources, high capacity for coordinated public health emergency management, cross-domain compensation, and timely multiple responses. Accordingly, these become the conditions for the urban agglomeration to resist the vulnerability of public health events. In accordance with previous research (53, 54) and composite measure dimensions of DPM (37), the research of vulnerability to public health events in urban agglomerations should focus on three aspects: risk pre-assessment, cross-domain risk compensation capability assessment, and multiple response mechanisms assessments. We defined PVUA dynamic evaluation system through three domains above that are important when preparing for, mitigating, and reducing the consequences of the public health events.

More specifically, based on the current surveillance systems of public health, increasing the efficiency and effectiveness of the public health system, the role of system stakeholders, the analysis and interpretation of surveillance data, and approaches to system monitoring and evaluation are crucial (55), sensitivity is adopted for risk pre-assessment in this research. The primary purpose of regional risk pre-assessment is to assess the probability that the event may occur before various emergencies arise, based on historical data and actual conditions (14). In the context of the types and characteristics of public health events in urban agglomerations, the scientific nature and availability of evaluation elements are considered. In this research, the potential risk factors in the pre-assessment of public health event risk in urban agglomerations are divided into social sensitivity, group sensitivity, and industrial sensitivity. Secondly, coping capacity is used for cross-domain risk compensation capability assessment. Risk compensation is a regional risk index obtained by considering the compensatory effects of institutions, medical and health resource allocation, and public health expenditures on reducing accident casualties based on inherent regional risks. Risk compensation has a defensive impact on regional risks. In this study, the cross-domain compensation capacity for public health events in urban agglomerations is constructed regarding the compensation capacity of rescue time and the compensation capacity of medical and health resource allocation. The cross-domain compensation capacity is resilient to the vulnerability of urban agglomerations to public health events, that is, reverse effects. Finally, the multiple response mechanisms are assessed by the level of collaborative governance (11). Building resilience needs adequate and committed investment in health through sustainable health financing (1). According to WHO, the total health expenditure is adopted as a quantitative indicator of total health investment, including government budget health expenditure, social health expenditure, and residents' health expenditure. The increase in budgetary, social, and residents' health expenditures will effectively reflect the government, society, and individuals' attention to public health events and the emergency response capabilities.

### Index system development

Based on the measure dimensions of PVUA dynamic evaluation system, by the complete consideration of comprehensive evaluation indices for the coping capability of urban emergency disasters (56), the comprehensive risk assessment model of multiple disasters (46), and the assessment model of urban security area (14), a research framework was constructed. Moreover, based on the basic framework of risk pre-assessment, cross-region compensation capability, multiple response mechanisms, and multi-agent collaborative governance, the PVUA is divided into three dynamic subsystems: sensitivity, coping capacity, and collaborative governance. These three interpenetrate and precisely concern the vulnerability reduction of public health events in urban agglomerations, which is a core specification element in the PVUA evaluation system.

Therein, the development of the urban economy and the aggregation of production factors enhance the sensitivity level; The coping capacity is determined by the medical system and medical services, which might mitigate public health events effectively; While regional collaborative governance capacity determines grassroots governance efficiency in coping with public health events. In this study, using scientific principles under consideration of operability, based on the characteristics of GBA and previous studies (14, 39, 46, 56–58), 23 third-level indices are selected to construct the evaluation index system of the PVUA for GBA (Table 1).

**Table 1:** Dynamic Evaluation index system of the PVUA in GBA.

**Dimension layer**	**Criterion layer**	**Factor layer**	**Index layer**	**Weight value**	**Index attribute**
Sensitivity (+) 39.36%	Social sensitivity	Population density	Resident population of the community/community area (SS1)/%	8.24%	positive
		Highway density	Highway mileage per unit area (SS2)/km	0.94%	negative
	Group sensitivity	Population mobility	Proportion of migrant population (PS1)/%	2.60%	positive
			Proportion of tenants in the community (PS2)/%	2.18%	positive
			Annual count of visitors (PS3)/10 thousands of people	4.35%	positive
		Population structure	Proportion of the population aged over 60 and below 14 (PS4)/%	1.88%	positive
	Industrial characteristic sensitivity	Industrial structure	Proportion of service sectors (IS1)/%	4.25%	positive
			Hoffman coefficient (IS2)/%	3.23%	negative
			Proportion of people engaged in agriculture (IS3)/%	1.96%	negative
		Opening degree	Foreign trade dependence (IS4)/%	9.74%	positive
Coping capacity (-) 43.63%	Risk compensation	Rescue time compensation capability	Proportion of areas with hospitals reachable within 5 minutes (TCR1)/%	4.77%	positive
			Proportion of areas with hospitals reachable within 10 minutes (TCR2)/%	3.95%	positive
			Proportion of 15-minute-travel rescue areas of hospital (TCR3)/%	3.75%	positive
	Risk response	Health resource allocation	Beds per thousand people (HCR1)	1.78%	positive
			Number of certified (assistant) doctors per thousand people (HCR2)	2.78%	positive
			Number of registered nurses per thousand people (HCR3)	2.66%	positive
		Health expenditure at all levels	Government budget for health expenditure (PRR1)/0.1 billion yuan	7.09%	positive
			Societal health expenditure (PRR2)/0.1 billion yuan	9.07%	positive
			Personal health expenditure of residents (PRR3)/yuan	7.78%	positive
Collaborative governance (-) 17.02%	Social governance of individual cities	Social governance	Coverage rate of Party organizations per community (SCG1)/%	2.05%	positive
			Number of neighborhood committees per community (SCG2)	5.19%	positive
			Number of village committees (SCG3)	5.69%	positive
	Multiple administrative principal units	Administrative efficiency	Number of sub-district offices (MGC1)	4.08%	positive

In the table, the symbol “+” indicates that the index is positive, and the symbol “-” indicates a negative index; the Hoffman coefficient is the ratio of light industry to heavy industry; foreign trade dependence describes the degree of openness of an economic system, foreign trade dependence = the total import and export value/GDP; Hoffman ratio (H') = industrial net output of consumption goods/industrial net output of capital goods; the area of rescue time compensation is obtained from GIS.

### Processing method of index data

Quantitative study and qualitative study are the typical research paradigms of social sciences. Currently, the studies on public security coordination in urban agglomerations mainly adopt the qualitative methodology. However, qualitative analysis often lacks persuasiveness (59, 60). Thus, more and more scholars began to propose and adopt quantitative methods to conduct research. In the evaluation method by the new build system, the entropy weight method and synthetic weighted index method are the most widely used methods to perform a quantitative evaluation, but data collected need to maintain a certain level of accuracy and comprehensiveness, which ensures the reliability of results. Accordingly, the data collected were further screened, cleaned, and subjected to normalization before data analysis.

A total of 23 index data of cities in GBA during the study period are normalized as follows.

Positive index:


(1) $$\begin{array}{r} Z_{ij}=\frac{\left(D_{ij}-D_{ijmin}\right)}{\left(D_{ijmax}-D_{ijmin}\right)} \end{array}$$

Negative index


(2) $$\begin{array}{r} Z_{ij}=\frac{\left(D_{ijmax}-D_{ij}\right)}{\left(D_{ijmax}-D_{ijmin}\right)} \end{array}$$

where *D*_*ij*_ is the normalized data value for the *i* index of the *j* city; *D*_*ijmax*_ is the maximum for the *i* factor of the *j* city; *D*_*ijmin*_ is the minimum for the *i* factor of the *j* city; *Z*_*ij*_ denotes normalized data.

According to the connotation of system vulnerability to public health events in urban agglomerations, the analysis is conducted in combination with the sensitivity-coping capacity-collaborative governance assessment framework. Since the effectiveness of sensitivity-coping capacity-collaborative governance on vulnerability is not equal, the weight value should be calculated according to the entropy method. The entropy method is an objective and scientific method to determine the weight, which can reasonably avoid subjectivity caused by artificial judgment. In combination with the entropy method, the determined weights are assigned under the indices of sensitivity, coping capacity, and coordination capability, and the system vulnerability degree of public health events in an urban agglomeration can be finally calculated.

In this study, the calculation of the PVUA is conducted with the third-level indices, so only the third-level indices are calculated in the index layer. First, the index of the dimension layer is set as *i*, and the third-level index layer is set as *j*, and then:


(3) $$\begin{array}{r} P_{ij}=\frac{{x^{{}^{\prime }}}_{ij}}{\sum_{i=1}^{m}{x^{{}^{\prime }}}_{ij}} \end{array}$$

In formula (3), *P*_*ij*_ denotes the weight of the *j* index under the *i* index; ${x^{{}^{\prime }}}_{ij}$ denotes the dimensionless index value for the *j* index under the *i* dimension.

Secondly, the entropy value *e*_*j*_ for the *j* index is calculated as follows:


(4) $$\begin{array}{r} e_{j}=\frac{-\sum_{j=1}^{m}P_{ij}\ln\;P_{ij}}{\ln\;m} \end{array}$$

Then, the information utility value *a*_*j*_ for the index is calculated.


(5) $$\begin{array}{r} a_{j}=1-e_{j} \end{array}$$

Finally, the weight *W*_*j*_ for the *j* index is calculated.


(6) $$\begin{array}{r} W_{j}=\frac{a_{j}}{\sum_{j=1}^{n}a_{j}} \end{array}$$

By using the utility value for the index information, and according to the additivity of entropy, the weight of the dimension layer *W*_*i*_ is calculated proportionally. By summing the weights of the indices of the determined index layer, *W*_*j*_, the weight of the index of the dimension layer *W*_*i*_ is obtained.

The synthetic weighted index method is used to conduct the weighted summation through each index to calculate the combined score for the synthetic evaluation. The evaluation model adopted for the synthetic evaluation value *Y*_*i*_ for each index in the criterion layer is (where $W=\;\frac{W_{j}}{W_{i}}$):


(7) $$\begin{array}{r} Y_{i}=\sum W{x^{{}^{\prime }}}_{ij} \end{array}$$

For the synthetic evaluation value *Y* of the target layer, the adopted evaluation model is:


(8) $$\begin{array}{r} Y=\overset{n}{\underset{j=1}{\sum }}W_{j}{x^{{}^{\prime }}}_{ij} \end{array}$$

In formula (8), when the target layer is sensitivity (positive index), the greater value for *Y* corresponds to higher sensitivity to regional public health events and greater vulnerability; when the target layer is coping capacity (negative index), the greater value for *Y* corresponds to higher regional coping capacity and less vulnerability; when the target layer is collaborative governance (negative index), the greater value for *Y* corresponds to higher regional collaborative governance capacity and less vulnerability.

The combined score Y is equal to the weighted sum of the scores of each item, and the formula is:


(9) $$\begin{array}{r} Y=Y_{1}+Y_{2}+Y_{3} \end{array}$$

In formula (9), *Y* is the synthetic score of the evaluated region, and *Y*_1_, *Y*_2_, and *Y*_3_ are synthetic scores of sensitivity, coping capacity, and collaborative governance.

### Calculation method of rescue time compensation capability

The rescue time compensation capability directly impacts regional risk compensation capability under major public health emergencies. It is closely associated with the distribution of medical stations and driving speed. In the case of emergency disasters and personnel first aid, “120” emergency vehicles are required to arrive at the site of the accident within the shortest time (generally within 5 to 15 min in central urban areas) to perform rescue services (58). Although geospatial analyses have been used to estimate health-care access in many countries, such techniques have not been widely applied to inform real-time operations in protracted health emergencies (61). Referring to the emergency response analysis method in the relevant literature (58, 61), considering the constraint of maximum speed in road traffic regulations and based on the *Technical Standard of Highway Engineering (JTG B01-2003, China)*, the road speed limits are set as 120 km/h for expressways, 80 km/h for urban expressways, 60 km/h for principal arteries, 50 km/h for secondary arteries, and 30 km/h for branch roads, respectively. Firstly, establish the factor data set, import data, verify the network data set, select the service area, and import service points accordingly. Secondly, the travel time for each road to the medical point was calculated using the formula, and fields were added to the attribute table. Thirdly, the impedance is set as a 5-min service area, 10-min service area, and 15-min service area to analyze the fastest path. Furthermore, according to the spatial analysis results, the ratio of compensation area to urban area is obtained to establish the data on rescue time compensation capability for each city.

## Temporal and spatial characteristics of sub-dimensions of the PVUA

### Determination of sensitivity level to public health events in GBA

**Figure 2:**
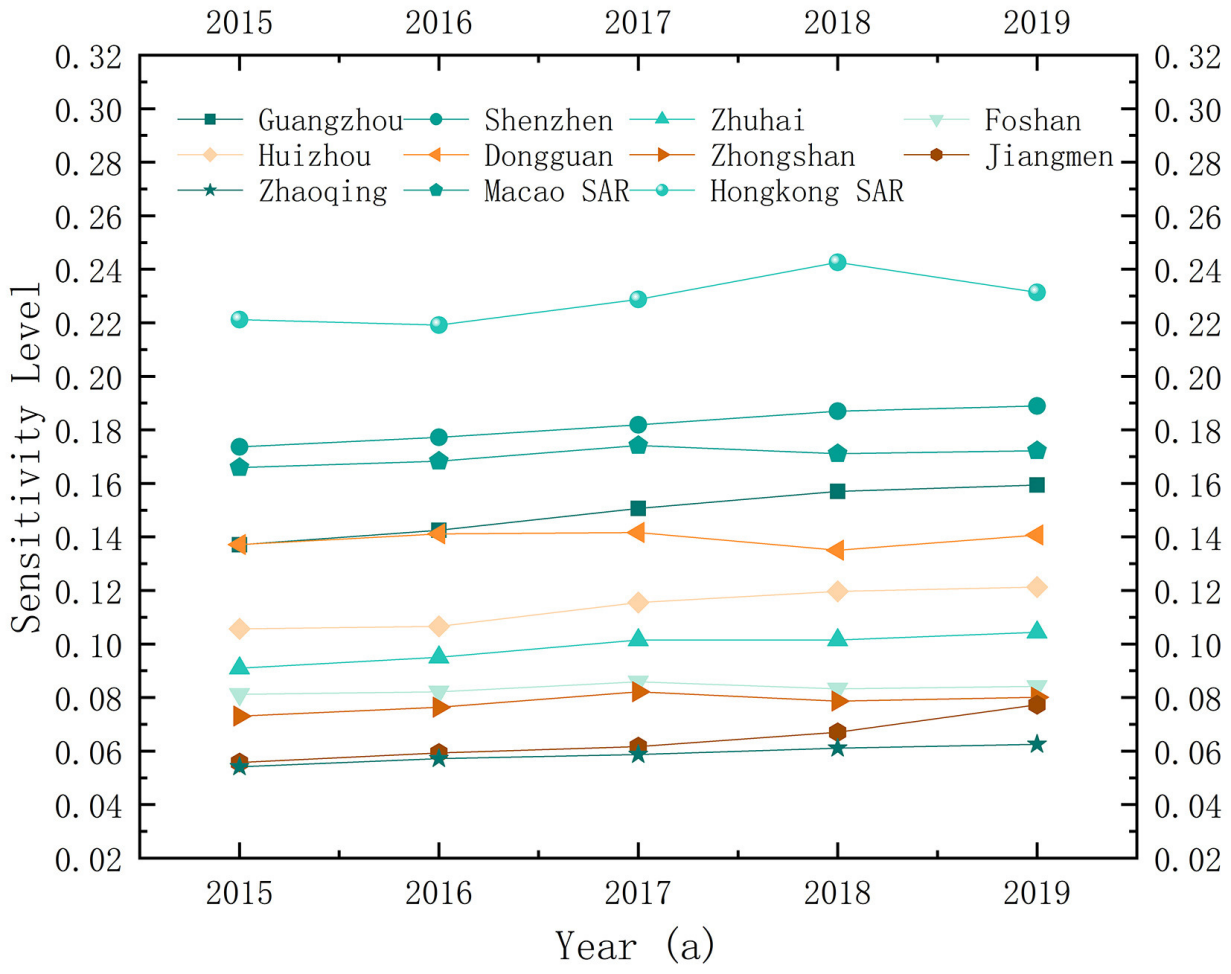
Sensitivity level in 2015-2019.

The weight of each normalized index is determined by the entropy method, and the sensitivity level of each city in GBA is calculated for each year. According to Figure 2, the sensitivity levels present a differentiated trend within GBA. During the study period, the sensitivity level of Hong Kong SAR is the highest. In detail, it declines, then rises, and finally declines again, constituting a fluctuating trend. As a possible reason, its foreign trade dependency is the highest and combined with the higher population density and the number of annual visitors, the sensitivity to the city's public health events is maintained at the highest level and is more significantly interfered with by internal and external social environments and the economic situation. Thus, Hong Kong SAR is exposed to a volatile situation. The sensitivity level to public health events is the second-highest in Shenzhen and constantly rises slightly because of its high population density and the constant increase in the number of visitors and the proportion of the service sector. Thus, the sensitivity to the influence of regional public health events increases together with the constant social and economic development of Shenzhen in the study period. Therefore, the occurrence of public health events on the city level deserves to be prevented.

Regarding the developing trend, the sensitivity levels of Guangzhou, Huizhou, and Jiangmen increased continuously, while that of Dongguan first decreased and then increased since 2016; the sensitivity levels of Macao SAR, Foshan, and Zhongshan followed an “inverted U” pattern of first increasing and then decreasing, and all cities reach their peak values followed by a gradual decrease in 2017 generally showing a relatively stable trend. Among them, the sensitivity level of Macao SAR remains in the high-value area due to the high degree of external dependency and its single-industry structure; however, with the transformation of Macao to a moderately diversified economic entity, the sensitivity level gradually decreased after 2017. The decrease in the sensitivity level of Foshan is mainly reflected in the transformation of its industrial structure (the Hoffman coefficient varies from 84.435 and 80.836% in 2016 and 2017 to 95.247 and 96.734% in 2018 and 2019, respectively). As a substantial manufacturing base in China, the sensitivity level of Foshan decreases due to the transformation of old to new driving forces and the high-quality development of the manufacturing industry in recent years. With the decrease in the external attraction of Zhongshan after 2017, the proportion of the migrant population and community tenants declines, and its sensitivity level decreases. The remaining cities are in a relatively stable state of fluctuation.

**Figure 3:**
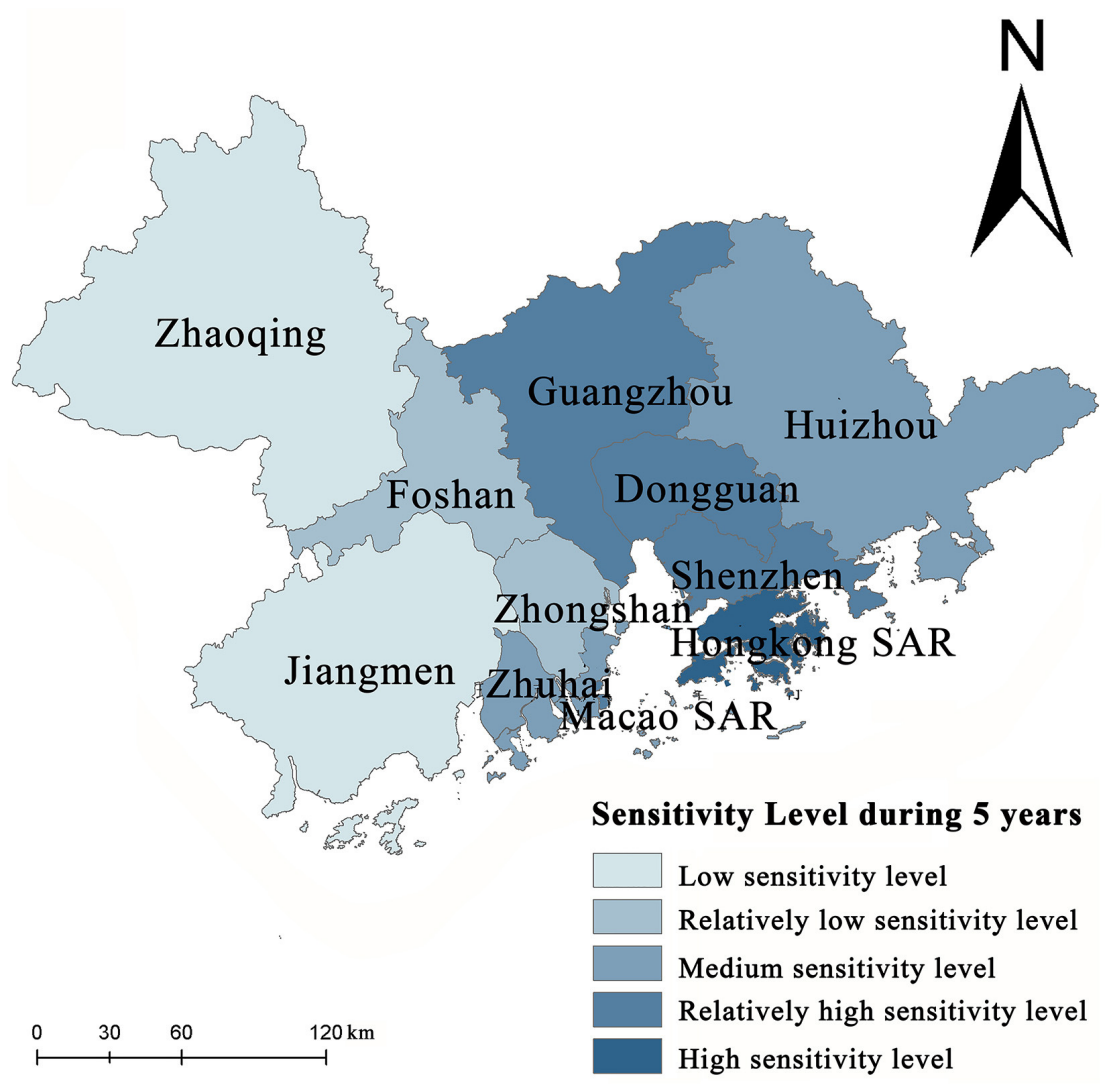
Spatial pattern of sensitivity level.

According to the natural breakpoint method, the sensitivity levels to the overall public health events in GBA during the study period are divided into five classes, as shown in Figure 3. According to the evaluation results of the spatial mapping, the sensitivity levels to public health events in GBA are the lowest in the western region, followed by the central region, and the highest in the southeast. With Hong Kong SAR and Macao SAR as the peak areas for sensitivity, the sensitivity decreases from Shenzhen, Dongguan, and Guangzhou to both borders of GBA, presenting a center-edge characteristic and the diffusion effect from the central cities to the surrounding cities is significant. In addition, Guangzhou, Shenzhen, Hong Kong SAR, and Macao SAR play the role of “leader geese” in industrial structure upgrading and transferring the upgraded industrial structure within the urban agglomeration in a “Flying Geese Model”. Since the sensitivity level to urban public health events is composed of social sensitivity, group sensitivity, and industrial characteristic sensitivity, the center of gravity range of labor force and population density expands with the development of the regional economy, the improvement of urbanization level, and the change of industrial structure, and contiguous agglomeration areas with high sensitivity levels are formed consequently. This horizontal spatial structure feature of the sensitivity level to public health events also proves from another perspective that the diffusion effect of the core layer of central cities in GBA is emerging, and the spatial flow of production factors and industrial factors in surrounding cities is constantly enhanced.

### Determination of the coping capacity level for public health events in GBA

**Figure 4:**
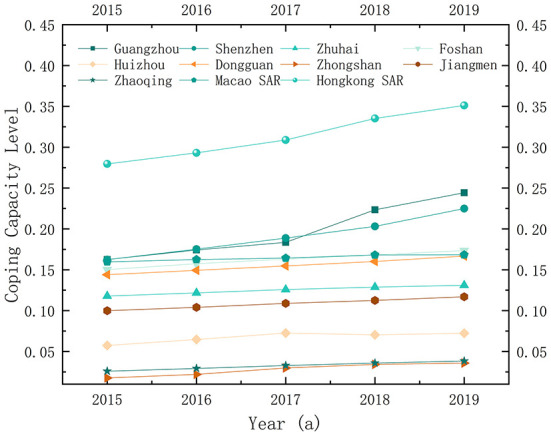
Coping ability in 2015-2019a.

According to the trend of the capacity to cope with public health events in GBA from 2015 to 2019, the coping capacity of all cities in GBA has improved to a certain extent (Figure 4), among which that of Hong Kong SAR, Guangzhou, and Shenzhen has increased by a relatively large margin. The overall medical and health service structures of the Hong Kong SAR are at the highest level, the area is relatively small, the emergency response area and the risk compensation area account for a relatively large proportion, and the capacity to cope with public health events is relatively high. As the core city of GBA and the capital city of Guangzhou province, Guangzhou has a relatively strong economic foundation and relatively complete medical service structure. Since the General Office of CPC Provincial Committee of Guangdong province issued the strategies for strengthening the capacity build-up of grassroots medical and health services in 2017, Guangzhou and Shenzhen have accelerated the construction of basic medical and health systems corresponding to a moderately prosperous society in all respects, comprehensively promoted the construction of a healthy Guangdong, increased the total amount of health care steadily, and enhanced the medical service capacity continuously. The remaining eight cities in GBA are in a stable fluctuation stage, and the compensation capacity and the coping capacity generally change little during the study period. Zhaoqing and Zhongshan show the lowest coping capacity, and it is necessary to improve the capacity of these cities to cope with public health events regarding the number of medical and health institutions and the coordination of transportation, the number of hospital beds per thousand population, the number of certified (assistant) doctors per thousand population, the number of registered nurses per thousand population, the ratio of doctors and nurses, and the health expenditure on residents by the government, by the society, and by individuals.

**Figure 5:**
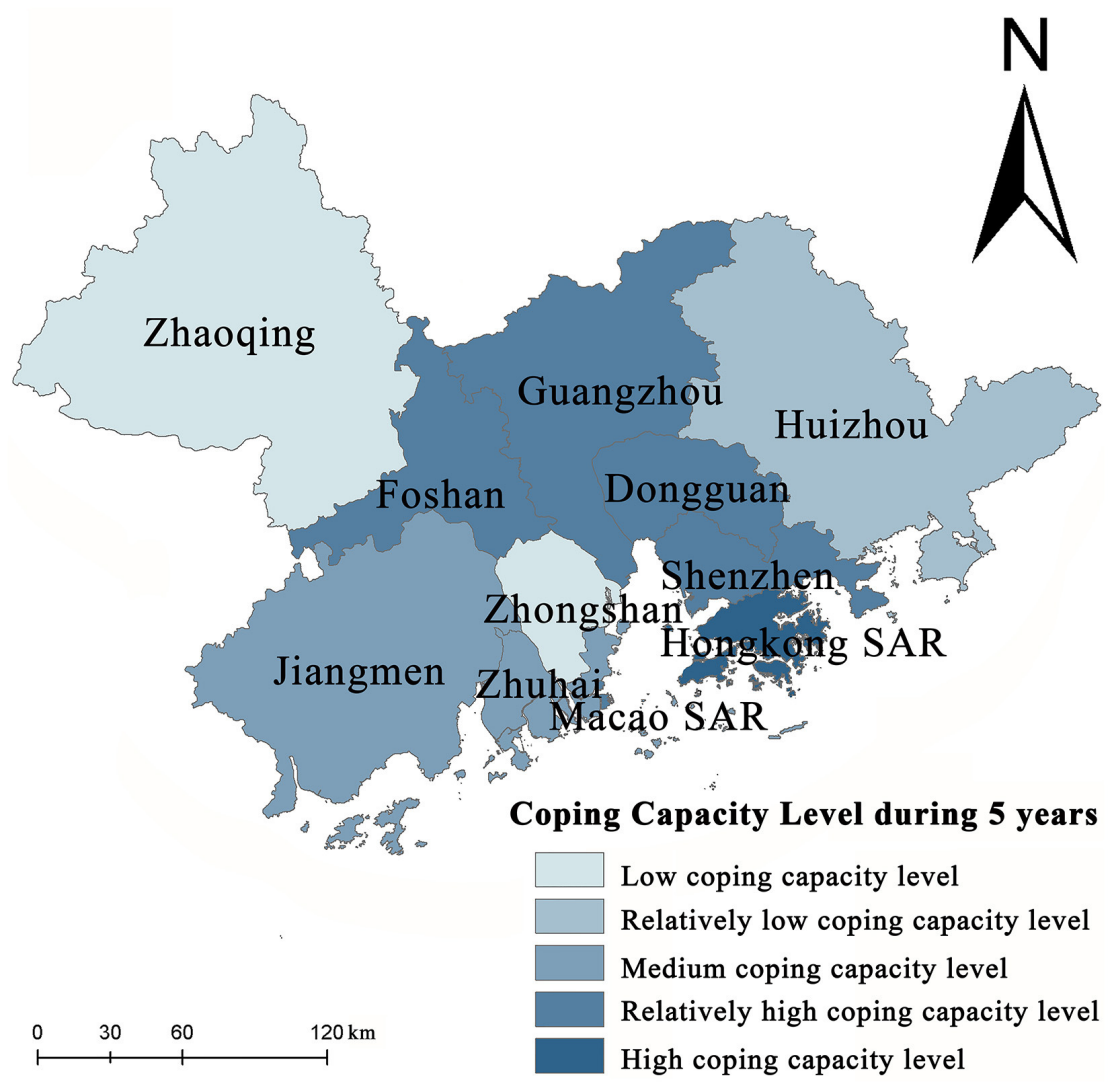
Spatial pattern of coping ability.

Spatial mapping of the capacity to cope with public health events in GBA from 2015 to 2019 was conducted, the areas in GBA characterized by different coping capacities were obtained, and a five-level division was performed according to the natural breakpoint method (Figure 5). At present, the coping capacity of various cities varies greatly in GBA. The overall spatial distribution shows a trend of being high in the southeast and low in the northwest. Hong Kong SAR, Macao SAR, Shenzhen, Dongguan, Guangzhou, and Foshan form a continuous area with high and relatively high coping capacity. According to Figure 5, coping ability is highly correlated with urban economic development. Therefore, it is of scientific significance to explain the coping capacity based on the urban economic development level. The diffusion effect and echo effect coexist in the spatial mechanism for the capacity to cope with public health events. In cities along the east bank of the Pearl River Estuary, this capacity presents a circle and layered structure caused by the diffusion effect. Hong Kong SAR and Shenzhen become the agglomeration area of coping capacity, spreading layer outwards by layer from the core, where the middle-high and middle-low level circles and layers are distributed successively. On the west bank of the Pearl River Estuary, Macao SAR, Zhuhai, Foshan, Jiangmen, and Zhaoqing also include Macao SAR as the core, showing a core-margin development trend. However, due to the relatively single-industry structure on the west bank of the Pearl River Estuary, sufficient driving force cannot be generated, and the “Myrdal turning point” has not been reached. Furthermore, the economic development of Zhongshan is at a lower level. As an inferior area, in the process of trade with surrounding cities and factor flow, talents, investment, population, and information are attracted to Foshan, Guangzhou, and Zhuhai, for which reason Zhongshan shows a backwash effect of the economy and coping ability.

### Determination of collaborative governance level to public health events in GBA

**Figure 6:**
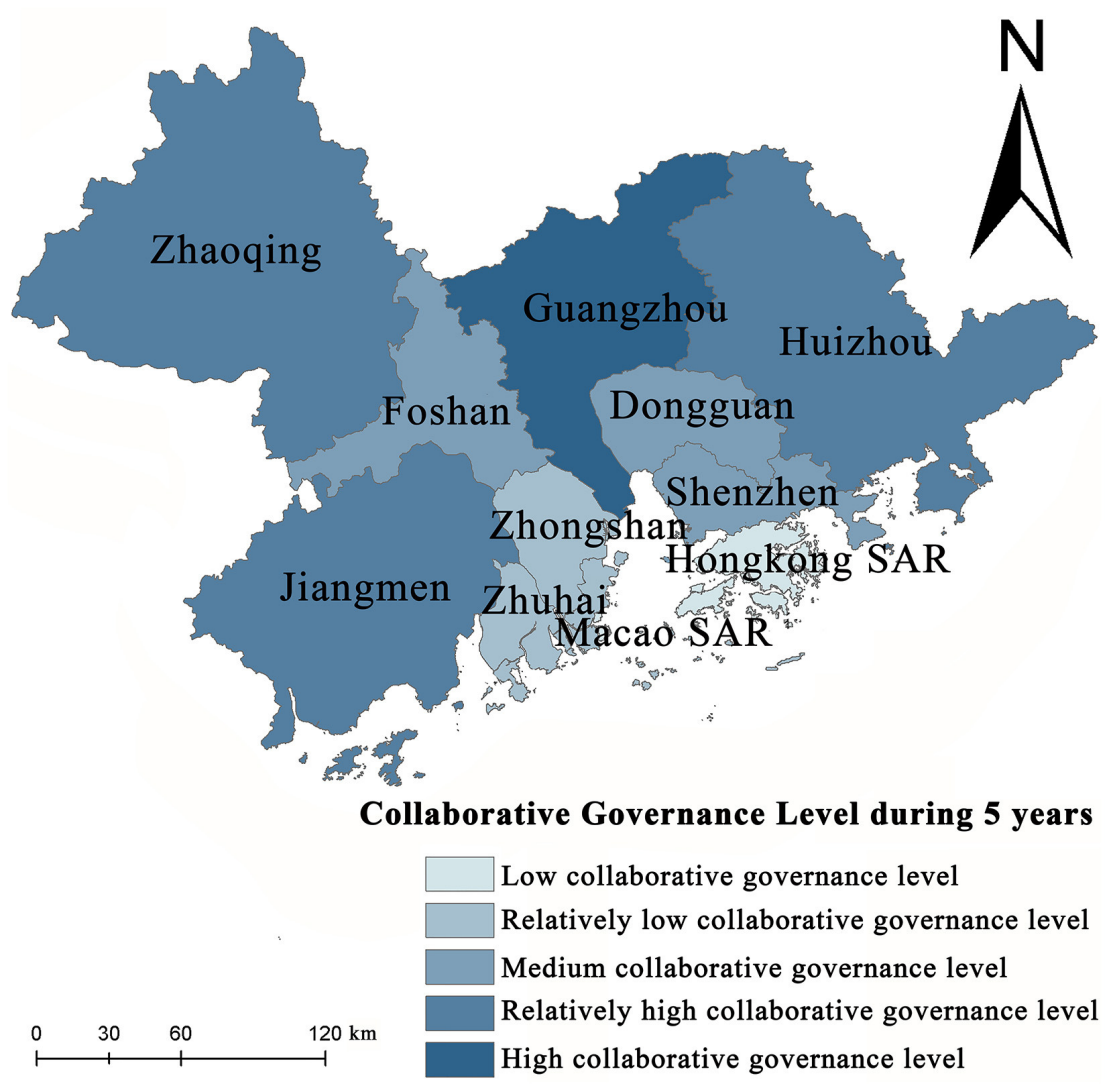
Spatial pattern of collaborative governance capability.

Cities in GBA have different capabilities for collaborative governance. According to the evaluation data, the collaborative governance capability is divided into five levels. Based on the analysis of the evaluation results (Figure 6), the collaborative governance capability in GBA is generally high, peaks in Guangzhou is low in the south, and is high in the north. The collaborative governance capability is low for Zhongshan and Zhuhai and shows its lowest levels for Hong Kong SAR and Macao SAR due to their differentiated social governance systems. Different from the sensitivity level and the coping capacity level associated with economic development, the collaborative governance capability of a city is measured based on social governance and administrative efficiency. As the provincial capital city, Guangzhou has high collaborative governance capability, while Dongguan, Shenzhen, Hong Kong SAR, Macao SAR, Zhuhai, and Zhongshan have formed contiguous areas with low collaborative governance.

## Temporal and spatial evolution characteristics of vulnerability to public health events

**Figure 7:**
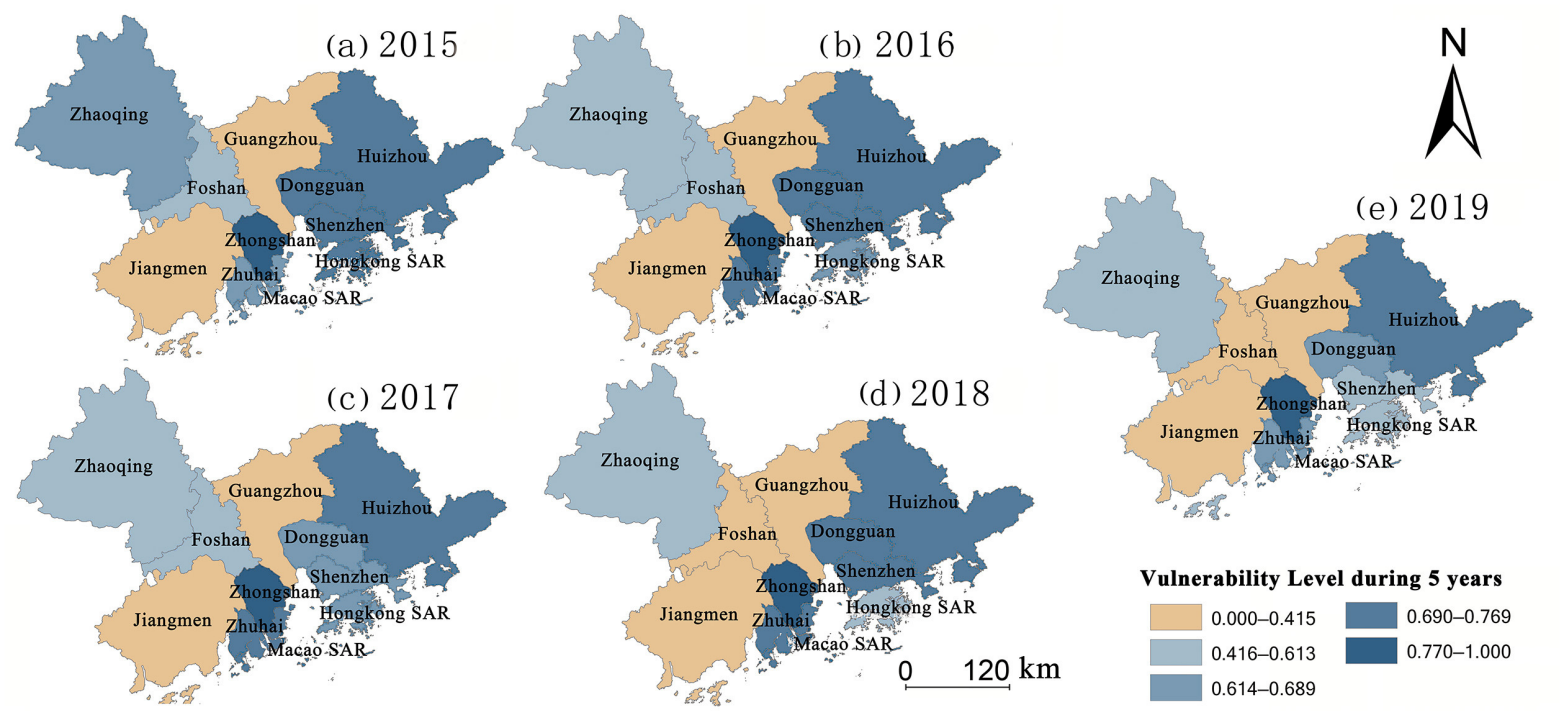
**(a–e)** Temporal and spatial evolution map of vulnerability level in GBA from 2015 to 2019.

Based on the above PVUA evaluation methods, the spatial pattern of five vulnerability types at the city scale in GBA was obtained by the natural breakpoint method in the study area during the 5 years. The study showed that the overall performance of the PVUA at the city scale in GBA was “low in the north and high in the south” during 2015–2019 (Figure 7). The results show that the volatility of vulnerability level reports gradually decreased, whereupon the comprehensive safe level gradually increased from 2015 to 2019. More specifically, the center of gravity of the highest level of city vulnerability shifted from northwest, northeast, and central of GBA (2015) to northeast and central of GBA (2017) to central of GBA (2019). The spatial pattern of vulnerability evolution of various cities in the GBA region changed drastically.

(1) Analysis of the node cities with the lowest levels of vulnerability in GBA

The lowest levels of vulnerability to public health events are found in Guangzhou and Jiangmen. Firstly, with the rapid economic development, the sensitivity level of Guangzhou presents a continuously increasing trend over the entire period (Figure 2). However, as the central city of GBA and the capital city of Guangdong province, Guangzhou has a robust policy environment for balancing economic development and enhancing the capacity to cope with the risk of public health events. In addition, with the improvement in medical and health resource reserves and medical service levels since 2017, Guangzhou's coping capacity has been rapidly improved (Figure 4), which balances the disaster-causing sensitivity factors such as highly concentrated production environments and population mobility that induce and spread public health events. Therefore, the regional vulnerability to public health events remains at the lowest level.

Furthermore, due to the stability of industrial characteristics and a smaller migrant population, Jiangmen has a low sensitivity level (Figure 2). Although the sensitivity level has increased since 2017 with Jiangmen becoming the main functional area for implementing industrial transfer, developing the equipment manufacturing industry, and establishing industrial supporting bases in Guangdong province, and because spatially Jiangmen, Guangzhou-Foshan metropolitan area, and the Shenzhen-Hong Kong-Macao economic circle constitute a “golden triangle” of GBA, Jiangmen is significantly characterized by its geographically advantaged position. The medical system and medical service capacity of Jiangmen are continuously improved, its coping capacity is improved at an accelerated level (Figure 4), and its vulnerability stabilizes at a low level.

(2) Analysis of the node cities with gradual decrease levels of vulnerability in GBA

Zhaoqing, Foshan, and Hong Kong SAR witness a gradual decrease in vulnerability to public health events. Among them, Zhaoqing and Foshan are at a low sensitivity level due to the characteristics of their industrial structure and low population mobility. In addition, the allocation of health resources and public health expenditure in the two cities are relatively high, and “medical insurance system, health system, and drug distribution co-reformation” are locally promoted, causing continuous improvement in coping capacity and the emergence of a declining trend of the vulnerability level. Hong Kong SAR has the highest sensitivity level, but its coping capacity is also at the highest level, and the allocation of health resources and public health expenditure constantly increases; thus, its vulnerability level gradually decreases. Furthermore, the vulnerability levels fluctuate in Shenzhen, Zhuhai, and Dongguan. During the study period, the collaborative governance capability of the three cities presents no significant change. At the same time, vulnerability fluctuates due to a correlation between sensitivity and coping capacity.

(3) Analysis of the vulnerable cities with a high level of vulnerability in GBA

The vulnerability to public health events remains high in Huizhou, Zhongshan, and Macao SAR. With the transformation of industrial structure and the continuous enhancement of population aggregation in these three cities, the industrial structure should be optimized in the future. Additionally, the number of medical systems, the allocation of health resources, and the public health expenditure should be increased to match the rapid development of the social economy in these cities. Although WHO guidance should be followed, a one-size-fits-all model will not be appropriate (62). Local government and GBA policy makers should execute targeted policies and actions are needed to protect the vulnerable cities with the greatest vulnerability.

Furthermore, available data suggest the vulnerability of public health events within the urban agglomeration posed by the combination of three factors: sensitivity, coping capacity, and collaborative governance capacity. Sensitivity is a positive factor, and coping capacity and collaborative governance capacity are negative factors for the vulnerability of public health events. According to the analysis of the spatial distribution of various elements in GBA, the spatial distribution of multiple components is inconsistent. For example, among the central cities in GBA, Guangzhou has a high sensitivity to public health events, and its coping ability and collaborative governance ability are high; while Shenzhen has high sensitivity, high coping ability, and medium level of collaborative governance; Hong Kong SAR and Macao SAR are cities with high sensitivity, high coping ability, but low collaborative governance ability; there are also small and medium-sized cities such as Zhaoqing and Jiangmen, which have low sensitivity, low coping ability, and relatively high collaborative governance ability. Based on this, strategy systems that deal with the risk of public health events should clarify the specific levels of various vulnerability factors first, thereupon formulating issue targeted policies to reduce system vulnerability.

In general, the overall evolution trend shows gradually expanding areas with low vulnerability, although the volatility of the vulnerability level varies significantly in GBA during the study period. In 2019, apart from Huizhou, Zhongshan, and Macao SAR, most of the cities in GBA fall into areas with moderate and low vulnerability levels (Figure 7e), and the capacity to withstand and cope with public health events continuously improved and achieved the highest level in GBA during the study period. As the reason for this improvement, with the release of the *Framework Agreement on Cooperation in Health and Wellness in GBA* in 2018 and the *Consensus on Cooperation in Medical and Health in GBA* in 2019, GBA focuses on complementing the advantages of each city or region and aiming at jointly building infrastructure and sharing benefits and continues promoting cooperation in high-quality medical resources, public health emergency response, innovation in traditional Chinese medicine, scientific research and services, talent cultivation, diagnosis, and treatment.

Although economic indicators are not used in the PVUA evaluation system, the study findings showed that the differences in regional economic levels expressed various vulnerability risks posed by public health events, which might uncover a hidden fact. More specifically, it is likely that the sensitivity level and coping capacity regarding the vulnerability to public health events in urban agglomerations are deeply correlated economic level or socioeconomic level inevitably, which is consistent with previous studies (54, 61, 63, 64). According to the research on Brazil denoted existing socioeconomic inequalities, rather than age, health status, and other risk factors for COVID-19, have affected the course of the epidemic (64). Firstly, the structural characteristics of population density, highway density, population mobility, industrial structure, and openness in economically developed areas lead to high sensitivity to public health events. Therefore, how coordinating the relationship between economic development and the risk of public health events becomes the key to reducing the vulnerability to public health events in GBA in the future. Secondly, with the social and economic development in a region and policy guidance, the proportion of rescue time compensation area, the allocation of health resources, and the levels of health expenditure at all hierarchies increase, which improves the internal capacity of urban systems to cope with public health events. Both the domain-specific and overall vulnerability index will help prioritize resource allocation in the face of constrained resources during the epidemic (35). In the future, the high coping capacity of Guangzhou should be exploited. A “one core, multiple points” medical network should be built with Guangzhou as the core. Available medical services can be expanded by creating traffic passages to provide more convenient and faster logistics and co-developed medical resource channels. Thus, the medical resource integration and the sharing of medical resources within the urban agglomeration in GBA can be achieved, and the overall coping capacity of GBA can be enhanced with the radiating and leading role of the central city.

Simultaneously, as articulated in The Lancet, 14 vulnerability in the present context is a dynamic concept—a person or a group might not be vulnerable at the beginning of the outbreak, but could subsequently become vulnerable depending on the government response (62). Effectively enhancing regional collaborative governance capability is the focus of work to reduce the vulnerability to public health events (6, 44) in GBA. Due to the differences in social management systems, the collaborative governance capabilities in Hong Kong SAR and Macao SAR have a significant disadvantage compared with other cities in the urban agglomeration, which further proves that the differences in market systems and barriers in factor flow are still considerable challenges faced by GBA to achieve coordinated development. Transforming “institutional barriers” into “institutional advantages” in GBA for cooperative management of public health emergencies, enhancing the collaborative governance capability of GBA, formulating a systematic collaboration framework, and promoting measures for standard cooperation schemes and emergency cooperation schemes are of great significance to reducing the risk of public health events in GBA.

## Conclusions and discussion

### Conclusions

In recent decades, with the advancement of urbanization in China, the aggregation and spatial mobility of production factors unique to urban agglomeration cause the safety security of public health events to be distinctly systematic, diversified, derivative, and intersectional. Accurate and effective measurement of vulnerability to regional public health events is most important to ensure people's health, economy, social stability, and political image in urban agglomerations (48). However, researchers have paid little attention to universal vulnerability system research on public health events in urban agglomerations. Its definition and connotation are not yet clear, and there is a lack of research on its antecedent influences and generation mechanism. Therefore, in this study, we expanded the DPM (37) in a Chinese urban agglomerations context, explore the structural dimensions of the PVUA dynamic evaluation system and its formation mechanism to expand and enrich the existing theoretical and applied research on managing public health events.

In this study, the spatial and temporal variation characteristics of the sensitivity-coping capacity-collaborative governance of vulnerability of city units in the study area were evaluated based on the PVUA model and GIS analysis (rescue time compensation capability), and entropy method during 2015–2019. Simultaneously, based on publicly available data, the temporal and spatial evolution characteristics of vulnerability to public health events in GBA were further explored in this research. The main conclusions are drawn as follows:

1. Population density, road density, population mobility, population structure, industrial structure, and openness are adopted as the six factors to assess the vulnerability to public health events in GBA. Surveillance is critical for improving population health (55). The sensitivity levels in Hong Kong SAR, Shenzhen, Macao SAR, and Guangzhou are relatively high. Regarding spatial distribution, the spatial sensitivity level pattern in GBA is characterized by the core-edge diffusion effect with the central city as the core. Furthermore, with the rapid economic development and aggregation of production factors, the sensitivity levels of multiple cities in GBA increase continuously.
2. The capacity of GBA to cope with public health events is assessed based on four factors: the rescue time compensation area, the allocation of medical and health resources, the public health expenditure, and the response of emergency supplies. According to the results, the spatial pattern of the coping capacity of the GBA presents a combined state of diffusion effect and echo effect; the coping capacity is found to be the highest in Hong Kong SAR, Guangzhou, and Shenzhen, reflecting continuous improvement in the medical and health system and the level of medical and health services with the social and economic development in these cities. The medical and health system, the construction of medical and health services, and the public health expenditure of Zhaoqing, Zhongshan, and Huizhou all belong to the lowest development area category, especially Zhongshan has become the core area of the echo effect.
3. As a provincial capital city, Guangzhou shows the highest collaborative governance capability in GBA, followed by Zhaoqing, Jiangmen, and Huizhou. The collaborative governance capabilities of Hong Kong SAR and Macao SAR are the lowest. In the future, in-depth cooperation based on “one country, two systems”, together with the *General Plan for the development of the Hengqin Guangdong-Macao In-depth Cooperation Zone* and *The plan for comprehensively deepening reform and opening up of the Qianhai Shenzhen-Hong Kong Modern Service Industry Cooperation Zone* in 2021, can promote more freedom and convenience in the flow of people, cargo, capital, and technology. Collaboratively handling public health events, improving the level of collaborative and coordinated management of public health emergencies, and reducing institutional barriers to cooperative management of public health emergencies have become the focus of work to reduce the vulnerability to the risk of public health events in GBA.
4. The vulnerability to public health events presents a significant declining trend in GBA, demonstrating the inevitable future trend of in-depth cooperation based on the construction of an encompassing high-quality living circle and the concept of excellent health in GBA, continuous enhancement of the capacity of GBA to withstand, handle, and collaboratively govern public health events, the achievement of gradual decline in the regional vulnerability to public health events, and joint development of social economy and medical care as well as public health.

## Discussion

In the present study, we developed and validated an dynamic instrument of the PVUA with good initial evidence for internal reliability. PVUA evaluation system was proved in virtual agreement with the actual situation in GBA. Although research on public health surveillance systems is relatively mature (55), to the best of our knowledge, the PVUA dynamic evaluation system represents the first instrument developed for urban agglomeration. According to the vulnerability principles of public health events, this study finds public health vulnerability assessment of urban agglomeration to be a complex process. Moreover, multiple angles and various methods should be adopted for the comprehensive factors analysis. The evaluation system could also contribute to increased stakeholders' awareness to identify the node cities of the vulnerability of public health events for the benefit of implementing a healthy China policy.

The study has some limitations that should be taken into consideration. First, we acknowledge that the PVUA dynamic evaluation system presented in the current study *via* previous research on public health policy and categories may not be prominent representative, and thus subsequent research might introduce more comprehensive research methods to demonstrate the current results. Second, there may be concerns about how the results would generalize beyond this sample to other urban agglomerations of China. Third, our data were based on a single evaluation system, and we assessed the geographical distribution features of the PVUA without conducting a reliability check. Future research might involve the adoption and use of methods in other health-related disciplines to provide evidence of synergistic interactions. Besides, the PVUA was tested in a Chinese urban agglomerations context; how it behaves in other cultural settings remains to be investigated.

Future research should also be deepened and expanded: regarding data processing, high-resolution satellite imagery, satellite data, big data of urban traffic should be introduced, and the data on rush hours, road accessibility and topographic elevation data should be fully considered to obtain more accurate rescue time compensation areas in each city. Moreover, horizontal research in this field is warranted. We invite future research to focus on more elaborate testing to evaluate the validity of the PVUA evaluation system, primarily to evaluate the applicability of the scale to different regions in other cultural settings. In addition, multiple comprehensive research for the PVUA evaluation system is also warranted.

## Data availability statement

The raw data supporting the conclusions of this article will be made available by the authors, without undue reservation.

## Ethics statement

The studies involving human participants were reviewed and approved by Hanshan Normal University Ethics Committee. The patients/participants provided their written informed consent to participate in this study. Written informed consent was obtained from the individual(s) for the publication of any potentially identifiable images or data included in this article.

## Author contributions

WC: conceptualization, visualization, writing-original draft, writing—review, and editing. JC: supervision, methodology, funding acquisition, and project administration. HS: supervision, investigation, data curation, and formal analysis. YaZ: validation and formal analysis. SL: data curation and investigation. YiZ: visualization and formal analysis. All authors contributed to the article and approved the submitted version.

## Funding

This research was funded by Guangdong Basic and Applied Basic Research Foundation (2019A1515110928), Guangdong Planning Office of Philosophy and Social Science (GD20CGL09), Hanshan Normal University, China (XN202031), Department of Education of Guangdong Province(2020GXJK091).

## Conflict of interest

The authors declare that the research was conducted in the absence of any commercial or financial relationships that could be construed as a potential conflict of interest.

## Publisher's note

All claims expressed in this article are solely those of the authors and do not necessarily represent those of their affiliated organizations, or those of the publisher, the editors and the reviewers. Any product that may be evaluated in this article, or claim that may be made by its manufacturer, is not guaranteed or endorsed by the publisher.
